# Cryostructuring of Polymeric Systems: 67 Properties and Microstructure of Poly(Vinyl Alcohol) Cryogels Formed in the Presence of Phenol or Bis-Phenols Introduced into the Aqueous Polymeric Solutions Prior to Their Freeze–Thaw Processing †

**DOI:** 10.3390/polym16050675

**Published:** 2024-03-01

**Authors:** Olga Yu. Kolosova, Viktor G. Vasil’ev, Ivan A. Novikov, Elena V. Sorokina, Vladimir I. Lozinsky

**Affiliations:** 1 A.N. Nesmeyanov Institute of Organoelement Compounds, Russian Academy of Sciences, Vavilov Street 28, Bld. 1, 119334 Moscow, Russia; kolosova@ineos.ac.ru (O.Y.K.); viktor@ineos.ac.ru (V.G.V.); 2 Prokhorov General Physics Institute of the Russian Academy of Sciences, Vavilov Street 38, 119991 Moscow, Russia; i.novikov@niigb.ru; 3 Microbilogy Department, Biology Faculty, M. V. Lomonosov Moscow State University, 119991 Moscow, Russia; evsorokina77@mail.ru; 4 Microbiology Department, Kazan (Volga-Region) Federal University, 420008 Kazan, Russia

**Keywords:** poly(vinyl alcohol) cryogel, phenols, physico-chemical properties, drug release, antimicrobial activity

## Abstract

Poly(vinyl alcohol) (PVA) physical cryogels that contained the additives of *o*-, *m*-, and *p*-bis-phenols or phenol were prepared, and their physico-chemical characteristics and macroporous morphology and the solute release dynamics were evaluated. These phenolic additives caused changes in the viscosity of initial PVA solutions before their freeze–thaw processing and facilitated the growth in the rigidity of the resultant cryogels, while their heat endurance decreased. The magnitude of the effects depended on the interposition of phenolic hydroxyls in the molecules of the used additives and was stipulated by their H-bonding with PVA OH-groups. Subsequent rinsing of such “primary” cryogels with pure water led to the lowering of their rigidity. The average size of macropores inside these heterophase gels also depended on the additive type. It was found also that the release of phenolic substances from the additive-containing cryogels occurred via virtually a free diffusion mechanism; therefore, drug delivery systems such as PVA cryogels loaded with either pyrocatechol, resorcinol, hydroquinone, or phenol, upon the in vitro agar diffusion tests, exhibited antibacterial activity typical of these phenols. The promising biomedical potential of the studied nanocomposite gel materials is supposed.

## 1. Introduction

The phenomenology of the freeze–thaw-induced sol-to-gel transformation of poly(vinyl alcohol) (**PVA**) aqueous solutions has been known since the 1970s [1,2,3]. The final “products” of such cryogenic processing (freezing—incubation in a frozen state—thawing) are gels that possess macroporosity and rubber-like elasticity [4,5,6]. In the early stages of their history, these materials were named by various terms, e.g., as “poly(vinyl alcohol) plastics” [2], “frozen poly(vinyl alcohol) gels” [7,8], “anomalous poly(vinyl alcohol) gels” [9], and “poly(vinyl alcohol) pseudo-gels” [10]. In 1984–1986 [11,12], the term “PVA cryogels” (**PVACGs**) was introduced into the scientific literature, and nowadays, such a word combination is most frequently used in relation to these gel matrices, their characteristics, and their applications [4,5,6,13,14,15,16,17,18,19,20,21].

Such physical (non-covalent) PVACGs are of a high practical interest in various fields, especially as materials for biomedical applications [4,5,16,18,19,20,21,22,23,24,25,26,27,28,29,30,31,32,33]. With regard to medical aspects, the use of cryogels based on PVA itself [19,20,22,28,32,33,34,35,36,37,38,39,40,41,42,43] and its blends with other (both synthetic and natural) polymers [18,44,45,46,47,48,49,50,51,52,53,54,55,56,57,58,59] and composite cryogels that contain entrapped functional disperse fillers [4,18,20,30,60,61,62,63,64,65,66,67,68,69] is currently considered as a very promising direction [19,20,22,23,25,26,27,28,29,30,31,32,33]. For instance, such materials are the non-toxic biocompatible carriers for drug delivery systems, e.g., in the form of temporary implants [70,71] or therapeutic coatings on wounds [40,42,49,54,64,67,68,69,72,73,74]. In this respect, it is of importance to have information both on the possible influence of a particular medication on the properties of the polymeric vehicle, PVACG in the present case, and on the peculiarities of drug release from this gel matrix.

Thus, when the molecules of a compound have ionic group(s), i.e., it relates to electrolytes, its incorporation into the initial PVA solution to then be gelled cryogenically can cause, depending on the chemical nature of this ionic substance, either salting-out or salting-in effects with respect to PVA [75,76,77,78,79]. In the former case, this can result in an increase in the rigidity of PVACGs thus formed. In contrast, in the latter case, the effect will more or less inhibit the gel formation and result in a decrease in the gel strength. Of course, the intensity of such effects will also depend on the solutes’ concentration. Analogously, both ionic and uncharged soluble additives capable of exhibiting kosmotropic properties (i.e., promote the PVA-PVA H-linking) cause the strengthening of the resultant PVACGs, whereas chaotropic additives (the substances that interfere with H-bonding) weaken the respective cryogels [80]. The main reason for such effects is due to the fact that the nodes of the spatial supramolecular network of these gel matrices are the zones of microcrystallinity, where neighboring PVA chains are connected by multiple intermolecular hydrogen bonds [4,5,6,81,82,83]. Therefore, the additives that exhibit positive or negative effects on the H-bonding are able to exert an influence on the physico-chemical properties and microstructure of PVACGs.

When water-soluble additives contain the hydrophobic fragment in the chemical structure of their molecules, this decelerates the kinetics of the solute release from the cryogel matrix owing to the hydrophobic interaction of such substances with the poly(methylene) core of PVA macromolecules [84]. In turn, when a hydrophobic moiety, e.g., a hydrophobic “tail”, acquires the surfactant properties to the respective soluble additive, its introduction into the initial polymeric solution will markedly influence the macroporous morphology (size and shape of large pores) of the resultant PVACGs because of a decrease in the surface tension at the interface between the growing ice crystals and the liquid phase in the course of initial system freezing [85].

**Figure 1:**
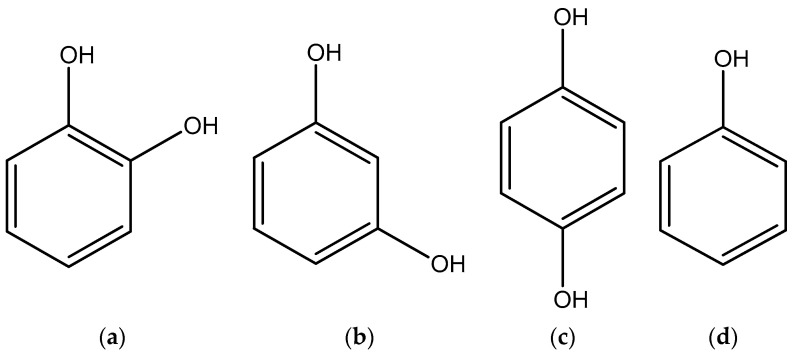
Chemical structures of pyrocatechol (**a**), resorcinol (**b**), hydroquinone (**c**), and phenol (**d**).

These examples evidently testify the need to know how additives of various chemical structure are able to interact with the PVA chains, to influence the cryotropic gel formation of this polymer, and, as a consequence, to affect the release behavior of substances entrapped in the cryogel bulk. The answer for these questions was the goal of the present study, where phenolic compounds, namely, *o*-, *m*-, and *p*-bis-phenols, as well as phenol itself, were used as water-soluble additives potentially capable of H-bonding with the OH-groups belonging to the PVA macromolecules [86,87,88]. It was also of interest to elucidate the effects of the interposition of hydroxyls in the molecules of bis-phenols in terms of their influence on the PVA cryotropic gel formation and the release of solutes from the respective PVACGs. In addition, as is well known, both various phenolic compounds and molecules containing phenolic structures (e.g., various antibiotics [89]) exhibit antibacterial and fungicidal properties and are used in the compositions of a number of medical and cosmetic products [90,91,92,93]. Therefore, PVA cryogels loaded with such phenolic additives can be considered as possible delivery systems for similar substances. In this context, in the present study, a series of PVACGs was prepared from the aqueous solutions of PVA containing bis-phenols such as pyrocatechol, resorcinol, and hydroquinone and also simple phenol (Figure 1) in varying concentrations. Then, physico-mechanical and thermophysical properties of the resultant cryogels were characterized, the macroporous morphology of the corresponding samples was studied, the kinetics of the phenolic compounds released from the gel matrix was investigated, and the antibacterial properties of cryogels loaded with the above-indicated phenols were evaluated in the in vitro experiments.

To the best of our knowledge, cryogels such as PVACGs formed in the presence of phenolic additives were earlier unknown, so their properties and microstructure and the features of the above-indicated solutes released from similar gel matrices have been studied for the first time.

## 2. Results and Discussion

### 2.1. Influence of Phenolic Additives on Rheological Properties of Aqueous PVA Solutions Used for the Preparation of PVACGs

As it was pointed out in the “Introduction”, various phenolic compounds are capable of H-bonding with the OH-groups of PVA macromolecules [86,87,88]. For this reason, already at the stage of preparation of the gel-forming polymer solutions that contained phenolic additives and were intended for the fabrication of the respective PVACGs, similar non-covalent interactions could affect the physico-chemical characteristics of such solutions and, as a consequence, influence the properties of the resultant cryogels. The examples of the exhibition of the discussed effects were the sol-to-gel transition and even polymer coagulation observed in this study after the introduction of certain amounts of phenol and bis-phenols into the PVA solution. Therefore, in the preliminary experiments, we initially revealed the concentration limits for phenolic additives when the respective polymer solutions remained liquid and could be used for further viscometry measurements. The lowest boundary concentration turned out to be no more than about 0.220 mol/L in the case of hydroquinone, and that is why this substance and other phenolic additives were further used up to a concentration of <0.2 mol/L.

**Figure 2:**
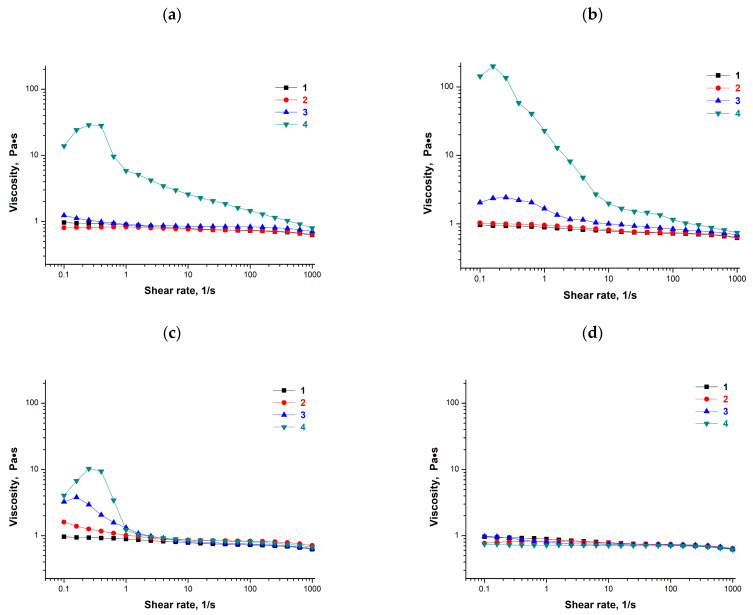
Flow curves for the 100 g/L aqueous PVA solutions without (**1**) and with the additives of pyrocatechol (**a**), resorcinol (**b**), hydroquinone (**c**), and phenol (**d**) in concentrations of 0.036 mol/L (**2**), 0.100 mol/L (**3**), and 0.180 mol/L (**4**).

The graphs in Figure 2 are the flow curves, i.e., the dependences of the dynamic viscosity versus the shear rate, for aqueous 100 g/L PVA solutions without and with the additives of various concentrations of *o*- (**a**), *m*- (**b**), and *p*-bis-phenols (**c**), as well as phenol (**d**). All these graphs are depicted at an equal scale in order to compare the respective curves under identical conditions.

It was found that all the tested polymeric solutions are non-Newtonian fluids, since their viscosity decreases more or less with the rise in the shear rate, i.e., the former property depends on the latter parameter. Thus, the flow of the PVA solution (curves **1** in Figure 2a–d) and the phenol-containing polymer solutions (curves **2** in Figure 2d) had a weakly expressed non-Newtonian character, while for the PVA solutions with the additives of bis-phenols, the non-Newtonian type of the flow was characterized by an extended structural branch in the flow curves (Figure 2a–c). With this, the spatial structures formed in such PVA solutions in the presence of pyrocatechol, resorcinol, or hydroquinone were destroyed under the influence of a mechanical field, i.e., at sufficiently high shear rates, the viscosities of these PVA solutions differ slightly from each other. The viscosity of the additive-free (curves **1** in Figure 2a–d) and the phenol-containing PVA solutions (curves **2**–**4** in Figure 2d) decreased slightly with an increase in the shear rate. Such a result testifies that this additive virtually did not affect the viscosity values and the shape of the flow curves, at least up to a phenol concentration of 0.18 mol/L. In turn, in the cases of effects caused by the introduction of increasing amounts of *o*-, *m*-, and *p*-bis-phenols into the PVA solutions, an increase in the viscosity was registered, especially at low shear rates (Figure 2a–c). This trend was most pronounced for the PVA solutions that contained the additives of resorcinol (Figure 2b). Therefore, it could be supposed that the *m*-location of phenolic groups in the resorcinol is able to provide a somewhat better geometric conformity for their hydrogen bonding with the OH-groups of neighboring PVA chains in the respective solutions compared to *o*- and *p*-bis-phenols. Hence, the features of the interactions of various phenolic additives with PVA in their mixed feed solutions should, as expected, influence the properties of PVACGs formed on the basis of such initial polymeric solutions.

### 2.2. Preparation and Physico-Chemical Properties of the Additive-Free and Phenolic-Additive-Containing PVA Cryogels

In this study, the physico-mechanical properties of the prepared PVACGs were evaluated using uniaxial compression tests (see Section 4.2.4). Therewith, the additive-free cryogels and the cryogels containing varied concentrations of phenolic additives were fabricated at two sub-zero temperatures (−20 and −30 °C) in order to trace the influence of this processing parameter on the properties of the resultant gel samples. This temperature range was chosen as a known condition for the formation of the PVACGs with the most well-reproducible characteristics [94,95].

**Figure 3:**
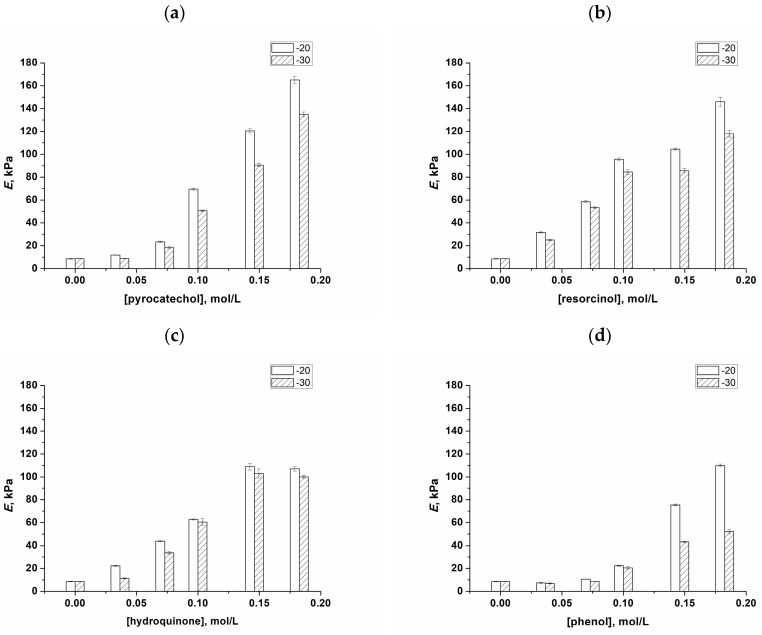
The values of the Young’s compression modulus (*E*) of PVACGs as dependent on the molar concentration of pyrocatechol (**a**), resorcinol (**b**), hydroquinone (**c**), and phenol (**d**) in the 100 g/L aqueous PVA feed solutions.

The obtained data on the values of Young’s compression moduli (*E*) for the cryogels of interest were collected and are shown in Figure 3, where the scales of the axes for the respective parameters are identical (for the sake of a better comparison) for all types of phenolic additives used in the experiments.

It was found that an increase in the concentration of *o*-, *m*-, and *p*-bis-phenols, i.e., pyrocatechol (**a**), resorcinol (**b**), and hydroquinone (**c**), respectively, as well as phenol (**d**), in the initial PVA solutions gave rise to the strengthening of the resulting PVACGs. This fact testifies both the participation of these phenolic compounds in the formation of 3D supramolecular networks in the course of PVA cryotropic gelation and the manifestation of the kosmotropic properties of these additives capable of promoting an additional interchain H-bonding. The latter conclusion takes into account the well-known cryoconcentrating phenomenon, i.e., a significant increase in the concentration of solutes during solvent crystallization upon freezing of the initial liquid system [4,96,97]. Therefore, we suppose that the concentration of additives in the unfrozen liquid microphase could reach a sufficient level for the manifestation of the kosmotropic-like influence of the respective phenols on the PVA-PVA association and, as a consequence, on the formation of PVA cryogels.

This effect was the most significant in the case of pyrocatechol-containing cryogels, when the *E* values of the PVACGs prepared in the presence of a 0.18 mol/L concentration of this bis-phenol (ortho-position of OH-groups) reached 140–170 kPa versus 8–9 kPa for the additive-free PVACGs (Figure 3a). This effect was somewhat lower for cryogels that contained resorcinol (meta-position of OH-groups) (Figure 3b) and hydroquinone (para-position of phenolic hydroxyls) (Figure 3c). Finally, this effect was lower in the case of gel samples prepared with the additives of simple phenol (Figure 3d); the latter cryogels we considered as the reference ones for comparison with the PVACGs containing different bis-phenols.

As for the influence of the temperature of cryogenic processing on the physico-mechanical properties of the additive-free PVACGs and PVACGs fabricated in the presence of phenolic compounds, the observed difference between the respective samples prepared at −20 and −30 Centigrade was not very significant (Figure 3) when the concentrations of added solutes were relatively low (<0.05 mol/L). At their higher concentrations, the PVACGs formed at −20 °C were more rigid compared to the cryogels prepared at −30 °C. The physico-chemical reason for this effect requires separate studies that were beyond the goal of the present research.

The data obtained (Figure 3) attest the key role of the chemical structure of bis-phenols, i.e., a relative position of phenolic hydroxyls in the aromatic ring of these substances, with respect to their influence on the spatial H-bonding with the OH-groups of PVA macromolecules. The order of the ability of these bis-phenols and phenol at concentrations as high as 0.1 mol/L to strengthen PVA cryogels turned out to be as follows: pyrocatechol > resorcinol > hydroquinone ≈ phenol. At the same time, if one compares this series with the order resorcinol > pyrocatechol > hydroquinone > phenol observed in the rheological experiments with respect to the influence of these solutes on the viscosity of the respective PVA solutions (Figure 2), one can see that the order of effects induced by *o*- and *m*-bis-phenols became the opposite. In other words, resorcinol additives increased the rigidity of PVACGs somewhat more strongly than equal concentrations of pyrocatechol (Figure 3), whereas the additives of the latter substance more strongly influenced the PVA association processes in the feed solutions, thus causing an increase in their viscometric parameters (Figure 2). We assume that one of the reasons for such a change in the trend after the cryotropic gelation of initial PVA solutions containing the additives of *o*- and *m*-bis-phenols is due to their distinct water-binding capacity. Whilst the solubility values of phenol and hydroquinone at room temperature are comparable and not very high (8.3 g and 5.9 g per 100 mL of water, respectively), the same parameters for the pair pyrocatechol/resorcinol are rather high and differ considerably (43 g and 110 g per 100 mL of water, respectively) [98]. These data mean that among such phenolic compounds, the highest affinity to water is inherent in resorcinol. Therefore, the volume of the unfrozen liquid microphase (the remaining small unfrozen portion of the macroscopically frost-solidified system [4,96,97,99]), where solutes are concentrated, will be higher in the case of resorcinol-containing samples in comparison to the pyrocatechol-containing ones, providing the freeze–thaw conditions are equal. In turn, the higher the volume of such liquid inclusions, the lower their PVA concentration and, as a consequence, the lower the rigidity of the resultant cryogel, since the less concentrated PVACGs commonly possess a lower gel strength [4,5,6,8,12,24,94,97,100,101,102,103].

**Figure 4:**
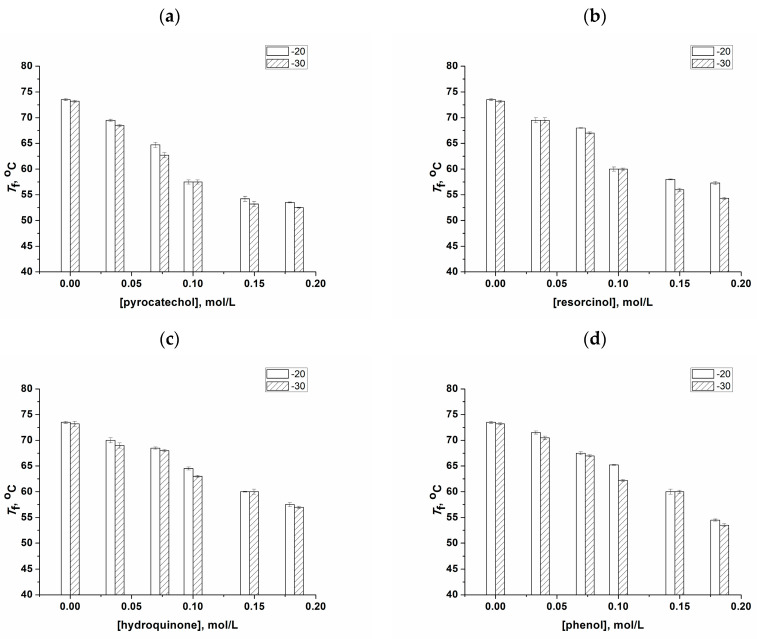
Fusion temperature values (*T*_f_) of PVACGs as dependent on the molar concentration of pyrocatechol (**a**), resorcinol (**b**), hydroquinone (**c**), and phenol (**d**) in the 100 g/L aqueous PVA feed solutions.

In this regard, it was also of significance to compare the heat endurance of similar cryogels in order to trace the exhibition of the common correlation usually observed for the freeze–thaw-fabricated physical PVACGs, namely, the higher their gel strength (e.g., elastic modulus), the higher their fusion temperature [4,94,104,105]. However, the respective experiments with the PVA cryogels prepared in the presence of phenolic additives quite unexpectedly revealed another trend. It turned out that the heat endurance of such cryogels progressively lowered with an increase in the concentration of these additives in the feed PVA solutions (Figure 4). Thus, the fusion temperature values of 73–74 °C inherent in the additive-free PVACGs fell down to 53–54 °C in the case of the pyrocatechol-containing cryogels (Figure 4a), to 54–57 °C for the resorcinol-containing gel samples (Figure 4b), to 57–58 °C for the hydroquinone-containing PVACGs (Figure 4c), and to 53–54 °C for the phenol-containing samples (Figure 4d)—with an additives concentration of 0.18 mol/L in all these cases.

On the one hand, the growth in the gel strength with an increasing content of phenolic additives in the resulting PVA cryogels (Figure 3) indicated an increase in the total amount of interchain cross-links within the supramolecular network of these PVACGs, thus giving rise to the formation of a denser matter of the gel phase (the walls of pores) in such heterophase macroporous polymeric matrices. However, on the other hand, simultaneous lowering of their heat endurance (Figure 4) indicated that the number of high-melting nodes in their supramolecular networks markedly decreased. It can be hypothesized that one of the reasons for such an effect is the action of added phenols as the H-bounding cross-linking agents that occupy some vacant OH-groups of PVA macromolecules, thus preventing their participation in the direct interchain PVA-PVA interactions. As a consequence, the total amount of cross-links increases with the rise in the phenolic additives’ initial concentration, but the fusion temperatures of the resultant PVACGs are lowered. This may be the case because stronger polarized H-bonds between the “acidic” phenolic hydroxyls and secondary alcoholic OH-groups [106] of the polymer are less energy-intensive in comparison to H-bonds in a pair of alcoholic hydroxyls belonging to the adjacent PVA chains.

In this respect, not only bis-phenols but even phenol itself, i.e., the mono-hydroxybenzene, are, in principle, able to form two or even three H-bonds with foreign OH-groups: one with the participation of a phenolic hydrogen atom and one or two with the participation of lone electron pairs belonging to the phenolic oxygen atom. In the case of the quinoid form of hydroquinone, H-bonds can be formed with two OH-groups of PVA. Nevertheless, the effect of reducing the heat endurance of the resulting cryogels with an increase in the concentration of phenolic additives (Figure 4) allows us to suggest that the dominant type of hydrogen bond capable of cross-linking the PVA chains in the systems under discussion is the above-mentioned interaction of acidic phenolic hydroxyls and alcohol OH-groups of PVA. The condition for the realization of all such cross-linking processes is the presence near the phenolic molecule of two sufficiently closely spatially spaced pendant hydroxyls belonging to neighboring polymeric chains. A definite argument in favor of such a mechanism can be the data further obtained on the physico-chemical characteristics of cryogels after they were washed with water from the phenolic additives, i.e., as a result of the transformation of “primary” PVACGs into “secondary” ones.

### 2.3. Changes in the Physico-Chemical Properties of PVA Cryogels as a Result of the Transformation of “Primary” PVACGs into “Secondary” Ones

**Figure 5:**
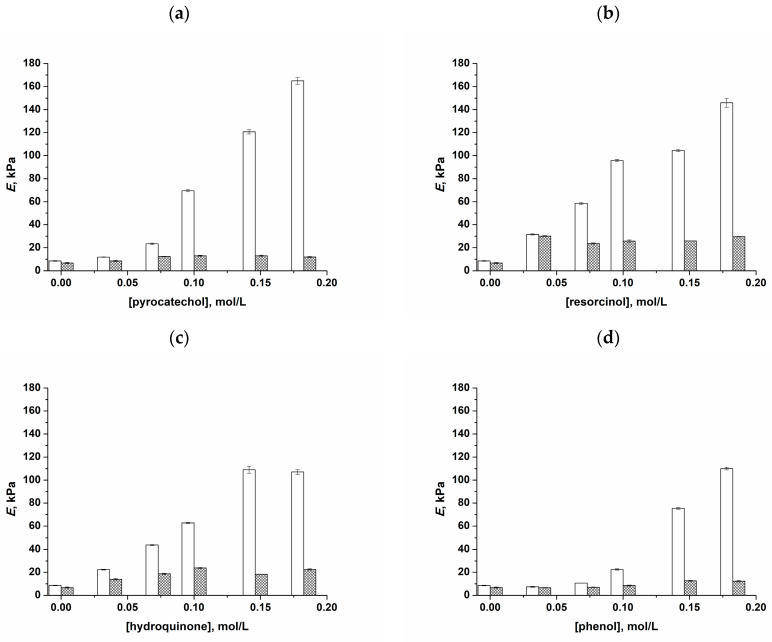
The values of the Young’s compression modulus (*E*) of the “primary” PVA cryogels formed at −20 °C (white columns) and the respective “secondary” PVACGs (grey columns) as dependent on the molar concentration of pyrocatechol (**a**), resorcinol (**b**), hydroquinone (**c**), and phenol (**d**) in the 100 g/L aqueous PVA feed solutions.

The samples of “primary” additive-free and additive-containing PVACGs all prepared by freezing at −20 °C were rinsed for 7 days with daily replaceable portions of deionized water (Section 4.2.4), after which the values of Young’s modulus were measured for the resultant “secondary” cryogels. These data are summarized as diagrams in Figure 5, where the values for the respective PVACGs before and after rinsing with pure water are compared.

It was found that the rigidity of the “secondary” additive-free PVA cryogels became insignificantly lower compared to the “primary” gel samples (Figure 5). Such an effect is known to be caused by some additional swelling of their supramolecular network as a result of washing out some sol-fraction of PVA [107,108]. In turn, the elastic modulus values of the “secondary” PVACGs derived from the additive-containing “primary” ones decreased considerably, from >100 kPa to 10–30 kPa, especially for those that were prepared in the presence of pyrocatechol (Figure 5a) and phenol (Figure 5d). This obviously means that the amount of interchain cross-links inside the water-rinsed PVACGs decreased considerably. Since simple rinsing with water is unable to break off the cooperative H-bonds within the microcrystallytes—the network nodes in the PVA cryogels—the above-indicated decrease in the amount of similar interchain non-covalent bridges relates to the replacement of weak cross-links in PVA–phenol(s) by the stronger H-bonds in PVA–water. Of course, this should result in a reduction in the cross-linking degree of the spatial network and, as a consequence, give rise to the lowering of the gel strength.

With this, the most rigid among the studied “secondary” PVACGs were the samples derived from the “primary” cryogels prepared with the additives of resorcinol (Figure 5b) and, to a somewhat lower extent, derived from the PVACGs fabricated with the additives of hydroquinone (Figure 5c). It is reasonable to assume that this effect was stipulated by the already-mentioned kosmotropic activity of the latter bis-phenols, when an increase in their initial concentration facilitated not only the coupling of the PVA–bis-phenol–PVA cross-links (those then were broken by water upon sample rinsing) but also promoted the formation of additional stable-in-water PVA-PVA hydrogen bonds.

### 2.4. Microstructure of the “Secondary” PVA Cryogels

It is evident that such an integral physico-mechanical index of the macroporous PVACGs as their Young’s modulus depends both on the elasticity of the proper polymer phase, i.e., the gel walls of macropores, and on the whole texture of these heterophase gel materials [4,12,14,94,95,101,109,110,111,112,113]. Therefore, in this study, the microstructure of the PVA-based cryogels was evaluated with the aid of environmental scanning electronic microscopy and using preliminary contrasting of the samples with neodymium-containing solution (Section 4.2.6). It was found that the latter process was effective only for the “secondary” cryogels, while in the case of “primary” PVACGs prepared with the additives of phenol or bis-phenols, these substances prevented the adsorption of such a lanthanide onto the polymeric matrix.

**Figure 6:**
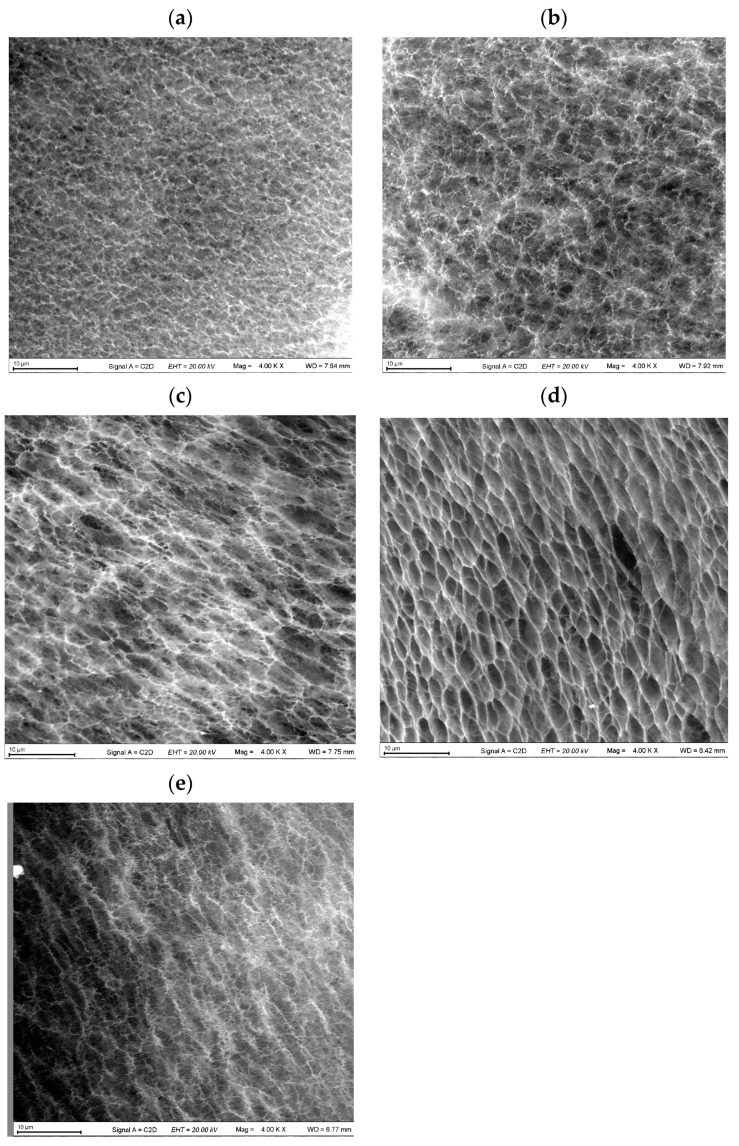
Environmental SEM images of microstructure of the “secondary” PVACGs derived from the “primary” cryogels that contained additives of pyrocatechol (**a**), resorcinol (**b**), hydroquinone (**c**), and phenol (**d**); the microstructure (**e**) of the “secondary” cryogel derived from the additive-free “primary” PVACG is given for the sake of comparison (scale bars—10 μm for all cases).

The SEM images thus obtained for the water-rinsed “secondary” cryogel samples are given in Figure 6. In these cases, the content of the respective phenolic substances in all the additive-bearing “primary” cryogels was 0.18 mol/L.

Rather evident distinctions in the macroporous morphology of these samples are well distinguished even at a qualitative level. Thus, the smallest pores are characteristic of the “secondary” PVA cryogel derived originating from the pyrocatechol-containing PVACG (Figure 6a), whereas a rather perfect ordered texture and the largest anisodiametric pores are inherent in the “secondary” cryogel derived from the “primary” PVACG formed in the presence of phenol (Figure 6d). The porous morphology of the sample in Figure 6c (cryogel after washing off hydroquinone additives from the respective “primary” PVACG) looks like a transitional one between the previous two gel matrices, and the pores in the “secondary” cryogel derived from the “primary” PVACG prepared with the additives of resorcinol (Figure 6b) are not so elongated in comparison to the samples in Figure 6c–e.

The average size of the macropores in the “secondary” PVACGs, whose texture is shown in the images in Figure 6, was measured using the Image J program (version 1.54h) (Section 4.2.6). The obtained pore size values are shown in Table 1.

Since the function of porogens in the course of PVA cryotropic gel formation fulfill the polycrystals of frozen solvent [4,14,21,85,94,95,99,100,109,110,111,112,113], one can say that the observed features of the microstructures of the considered PVACGs indirectly, at least, illustrate the influence of various phenolic additives on the size of the shape of ice crystals being formed upon the freezing of the corresponding PVA solutions.

For instance, the comparison of the pore size data (Table 1) and the gel strength of “primary” PVACGs (Figure 3), i.e., the heterophase gel matrices just after the formation of their macroporosity, reveals that the most rigid were the cryogels prepared with the additives of pyrocatechol (Figure 3a), and exactly these PVACGs (Figure 6a), among the other ones (Table 1), had the smallest pores, i.e., possessed a lesser structural defectiveness. However, for the rest of the cryogels considered in this study, no such explicit structure–properties correlation was found, most likely because of the unequal mechanisms of the influence of different factors on the characteristics of the cryogels formed as a result. Such factors undoubtedly include the already-mentioned significantly different solubility of phenolic additives in water and their different promoting effects on hydrogen bonding, as well as others, which have not yet been established.

### 2.5. Peculiarities of Phenolic Substance Release from the Additive-Containing “Primary” PVA Cryogels

In the “Introduction” of this paper, the high application potential of PVA cryogels as carriers for delivery systems for drugs and antimicrobial and cosmetic compounds was pointed out [33,34,35,36,37,114]. Therefore, we tested the PVACGs of interest in relation to the features of bis-phenols and phenol release from the respective “primary” cryogels prepared in the presence of such additives. These experiments were carried out both (i) as the simple diffusion of the respective phenolic compounds from the cryogel vehicles to the neat water (Section 4.2.7) and (ii) as the release of the same phenols from the cryogel carriers to the microbial mats for the evaluation of the bactericidal action of the systems “PVACG—phenolic additive” towards microbial cells (Section 4.2.8). All these tests were performed using the cryogel samples that contained the respective phenols at a concentration of 0.18 mol/L.

**Figure 7:**
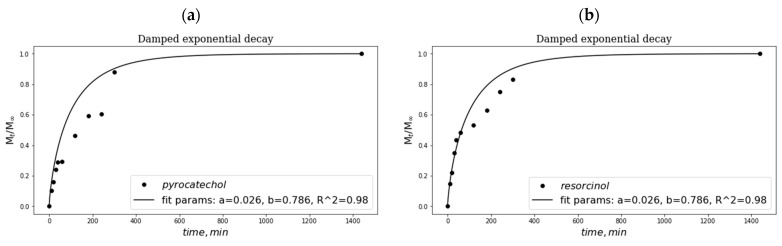

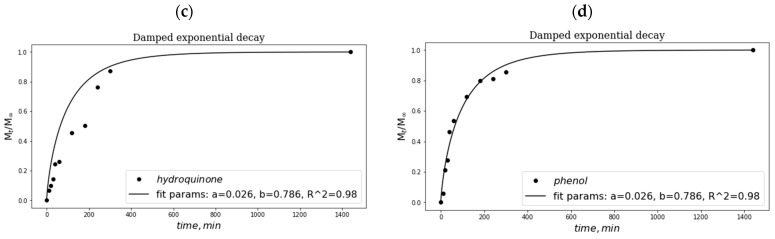
Kinetic profiles of the release of phenolic additives (pyrocatechol (**a**), resorcinol (**b**), hydroquinone (**c**), and phenol (**d**)) from the respective “primary” PVACGs.

The results obtained for the case (i) are given below in Figure 7 as the release kinetics curves depicted in the coordinates of the Weibull’s function [115]:

*M*_t_/*M*_∞_ = 1 − exp(–*a* × *t^b^*),

where *M*_t_/*M*_∞_ is the solute fraction released from the matrix for time *t*; the parameters *a* and *b* are the constants. The latter values (given in the framed insertions in each graph of Figure 7) were calculated using the ORIGIN PRO (version 15) software (OriginLab Corp., Northampton, MA, USA) by uploading the Weibull’s equation and the experimental data to this computer program. According to refs. [116,117], the constant *b* in this equation is a descriptor related to the influence of the gel matrix structure on the drug release. In this context, the *b* parameters within their values of 0.605–1.029 inherent in the studied systems testify to the virtually free diffusion mechanism [118] for the release of all used phenols from the macroporous matrix of PVA cryogel.

The comparison of the rates of this phenolic compound release at its early stage, e.g., for the first 300 min, revealed the following sequence: resorcinol (Figure 7b) ≈ phenol (Figure 7d) > pyrocatechol (Figure 7a) > hydroquinone (Figure 7c). Evidently, such an order is stipulated by the intensity of the dissociation of the polarized H-bonds [106] between the OH-groups of PVA and phenolic hydroxyls belonging to each particular phenol entrapped in the gel walls of macropores in PVACGs.

**Figure 8:**
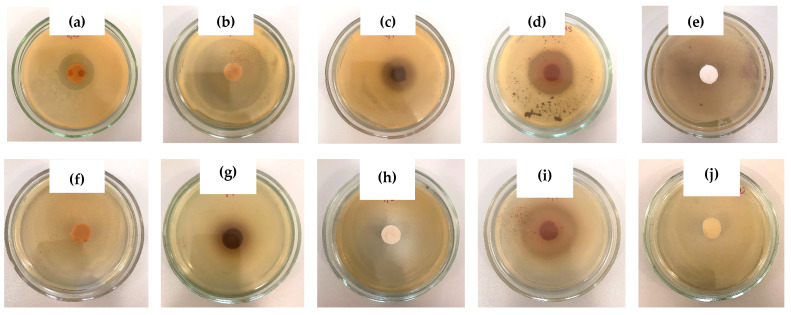
Growth inhibition zones formed around the PVA cryogels that contained 0.18 mol/L of pyrocatechol (**a**,**f**), resorcinol (**b**,**g**), hydroquinone (**c**,**h**), and phenol (**d**,**i**) or were additive-free (**e**,**j**) after sample incubation for 24 h onto the lawns of *S. aureus* (**a**–**e**) and *E. coli* (**f**–**j**) bacterial cells.

The efficient release of *o*-, *m*-, and *p*-bis-phenols, as well as phenol, from the carriers based on PVA cryogel was been confirmed when the bactericidal activity of these systems was tested with the agar diffusion technique (Section 4.2.8). Examples of the respective agar dishes are shown in the photographs in Figure 8, where the so-called “growth inhibition zone” (GIZ) [119] was formed around the PVACG loaded with different phenolic additives after the 24 hrs long incubation of the cryogel samples onto the microbial lawns of *Staphylococcus aureus* (Gram+) and *Esherichia coli* (Gram−) bacterial strains. Additionally, the quantitative data on the GIZ diameters for the cultivation for 24 and 48 h are summed up in Table 2. With this, the GIZ diameters higher than 30 mm testify to a rather high antimicrobial activity of the respective additive-containing cryogel samples.

First of all, it was observed that the growth of the Gram-positive *S. aureus* bacteria was inhibited by all the used phenols (Figure 8a–d; Table 2), at the highest extent by the resorcinol-containing PVACG (Figure 8b; Table 2). In turn, the Gram-negative *E. coli* cells had low sensitivity to the action of pyrocatechol- and hydroquinone-containing samples (Figure 8f,g) virtually analogously to the absence of any bactericidal influence of the additive-free PVACGs (Figure 8e,j). Principally, similar features of the antimicrobial activity specificity of various bis-phenols are known [120,121] and, evidently, should be taken in account upon the possible application of the respective loaded PVACGs as biomaterials.

In any case, the experimental results described in this section clearly confirm the efficient release of phenolic additives from the PVACG-based macroporous carriers. A similar release mechanism of various substances from the PVACG-based matrices was also observed for the diverse drugs (e.g., see refs. [122,123,124,125,126,127,128,129]), thus confirming the promising potential of PVA cryogels as polymeric vehicles for drug delivery systems.

## 3. Conclusions

Poly(vinyl alcohol)-based physical gel matrices prepared using a cryostructuring approach, i.e., the so-called PVA cryogels, are of significant scientific and applied interests, especially as materials for biomedical applications, e.g., as macroporous carriers in drug delivery systems. In this context, it is necessary to understand how various foreign additives, when these are present in the gel matrix according to the medical requirements, will affect the physico-chemical properties of the respective PVACG. With this, it is also of significance to know how the polymeric phase itself is able to influence the drug release processes. Taking these considerations into account, we have studied the influence of varied concentrations of phenolic additives, namely, *o*-, *m*-, and *p*-bis-phenols (pyrocatechol, resorcinol, and hydroquinone, respectively), as well as phenol itself, on the viscometric characteristics of the initial PVA–phenol solutions and then on the rigidity, heat endurance, and microstructure of the PVA cryogels formed as a result of the freeze–thaw processing of such solutions. In addition, the release behavior of the same phenolic compounds from the cryogel-based carriers has been examined with both kinetic experiments and microbiological tests. The main effects revealed in the respective experiments were as follows:

(i) The introduction of increasing amounts (from 0.036 to 0.180 mol/L) of bis-phenols in the PVA solutions resulted in the growth of their viscosity, thus indicating the hydrogen bonding of the additives with the OH-groups of neighboring PVA chains. This trend was mostly pronounced for the PVA solutions that contained the additives of resorcinol (*m*-location of phenolic groups in its molecule). The additives of phenol within the same concentration range did not exert a marked influence.

(ii) Subsequent cryogenic processing of such additive-containing PVA solutions resulted in the formation of the “primary” PVA cryogels whose Young’s compression moduli and fusion temperatures were dependent on the additive’s type and concentration. All the additives caused an increase in the gel strength, while the heat endurance of the gel samples was lowered. It is supposed that this trend is stipulated by differences in the thermal stability of interchain H-bonds between the alcoholic OH-groups of PVA against the stability of polarized H-bonds between the “acidic” phenolic and alcohol hydroxyls, when the molecules of the additives act as cross-linkers for the neighboring PVA macromolecules.

(iii) Rinsing of the additive-containing “primary” cryogels with pure water gave rise to the “secondary” PVACGs whose rigidity turned out to be significantly lower as compared to that of the “primary” additive-containing cryogels. And again, the values of the Young’s modulus for the “secondary” samples depended on the type and concentration of phenolic additives that were introduced into the initial PVA solutions prior to their cryogenic processing.

(iv) SEM studies revealed the morphological peculiarities of the water-rinsed PVACGs, where the largest macropores (4.02 ± 0.52 μm) were inherent in the “secondary” cryogels derived from the “primary” samples prepared in the presence of phenol and the smallest macropores (1.70 ± 0.24 μm) were present in the “secondary” cryogels derived from the PVACGs formed with the additives of pyrocatechol.

(v) The character of the phenolic additive release from the respective “primary” PVA cryogels was examined as a simple diffusion of the solutes from the cryogel vehicles to the neat water and as the release of the same phenols from the cryogel carriers to the microbial mats of two test bacterial strains: *Staphylococcus aureus* (Gram-positive) and *Escherichia coli* (Gram-negative) cells. These experiments showed a virtually free diffusion mechanism for the release of all used phenols from the macroporous matrix of PVA cryogel and, as a consequence, demonstrated the efficient antibacterial properties of such nanocomposite delivery systems.

(vi) In general, we have revealed the influence of the interposition of hydroxyls in the molecules of bis-phenols introduced into the initial PVA solutions prior to their freeze–thaw processing on the properties of the resultant cryogels and the release of these solutes from the respective PVACGs.

Hence, when certain water-soluble drugs include some bis-phenol-like fragments, and these substances are incorporated into the matrix of a PVACG-based carrier for the system application as a drug delivery biomedical nanocomposite material, the effects revealed in this study should be taken into account.

## 4. Materials and Methods

### 4.1. Chemicals

The following substances and reagents were used in the experiments as purchased: poly(vinyl alcohol) with a molecular weight of ca. 86 kDa and a deacetylation degree of 99–100% (Acros Organics, Geel, Belgium), phenol (99.5%, Reakhim, Moscow, Russia), resorcinol (99,4%, Eco-tec, Samara, Russia), hydroquinone (99–100%, Spectr-Chim, Moscow, Russia), and pyrocatechol (99%, Alfa Aesar, Ward Hill, MA, USA). The neodymium-containing contrasting solution for scanning electron microscopy (SEM) was from the BioREE-A kit produced by Glaucon LLC (Moscow, Russia). Deionized water was used for the preparation of aqueous solutions.

### 4.2. Methods

#### 4.2.1. Preparation of the Initial PVA and PVA/Phenol Solutions

In total, 10 g of PVA powder was dispersed in 100 mL of water and left overnight for swelling of the polymer followed by its dissolution by heating the mixture in a boiling water bath for 1 h. The sample was weighed before and after heating, and the amount of evaporated water was compensated for so that the resultant PVA concentration was 100 g/L. Then, the required amount of the respective phenolic compound was added and dissolved at room temperature. The concentrations of such additives were varied from 0.0365 to 0.20 mol/L.

#### 4.2.2. Viscosimetry Studies of the Additive-Free and Additive-Containing PVA Solutions

Dynamic viscosity (*η*, Pa·s) of the aqueous polymer solutions was measured at 20 °C over a range of 1–1000 s^–1^ of shear rates using a Physica MCR-302 rheometer (Anton Paar, Graz, Austria) equipped with the automatic gap control system Tru-Gap^TM^. Measuring plate–plate cell was of 50 mm in diameter.

#### 4.2.3. Preparation of the Additive-Free and Additive-Containing PVA Cryogels

PVACGs for the physico-mechanical measurements and for fusion temperature testing were formed in sectional duralumin cylindrical molds (inner dia. 15 mm, height 10 mm). The molds were placed into the chamber of precision programmable cryostat FP 45 HP (Julabo, Seelbach, Germany), where the respective PVA solutions were frozen and incubated at −20 °C or −30 °C for 12 h, followed by the defrosting of the samples upon the temperature rising to 20 °C at a rate of 0.03 °C/min governed by the cryostat microprocessor.

#### 4.2.4. Physico-Mechanical Characteristics of the Additive-Free and Additive-Containing PVA Cryogels

The Young’s compression modulus values (*E*) for the PVACG samples were measured using a TA-Plus automatic texture analyzer (Lloyd Instruments, Bognor Regis, Wesr Sussex, UK) from the linear portion of the stress–strain dependence, as described elsewhere [34,43,44]. The uniaxial loading rate was 0.3 mm/min, and testing was conducted until 30% deformation of the cryogel samples was reached. These physico-mechanical measurements were carried out at 21 ± 1 °C. Three parallel samples were examined in three independent experiments. The results thus obtained were averaged using Excel 2010 software.

In addition, some of the prepared PVA cryogels were placed in 100 mL of pure water and were incubated there for 7 days; periodically, pure water was refreshed for every sample till the absence of characteristic UV absorbance spectrum in the rinsing water. After that, the values of the Young’s modulus and fusion temperature (see Section 4.2.5) were measured for such water-rinsed samples.

#### 4.2.5. Fusion Temperatures of the Additive-Free and Additive-Containing PVA Cryogels

The heat endurance of the cryogel samples of interest was evaluated via the measurement of the gel fusion temperatures (*T*_f_) using the falling-down ball procedure, in detail described for PVACGs in [71]. For this aim, a cylindrical sample of PVACG was placed in a plastic tube; thereafter, a shallow incision was made on the upper surface of the cryogel with a scalpel, followed by the insertion a stainless-steel ball with a diameter of 3.5 mm and a mass of 0.275 ± 0.005 g. The tube was tightly corked and placed in a water bath equipped with a stirrer. The bath was heated at a rate of about 0.4 degrees/min. The temperature at which the ball, passing through the fused gel layer, fell to the bottom of the test tube was taken as the *T*_f_ of the sample.

#### 4.2.6. Microstructure of PVA Cryogels Formed in the Absence and in the Presence of Phenolic Additives

The microstructure of the prepared PVA cryogels was investigated with the environmental SEM technique. The following protocol was used for the preparation of samples for the SEM studies. The additive-free and the additive-containing PVACGs were placed in cylindrical silicone tubes (inner dia. 3 mm), followed by their flash freezing in liquid nitrogen. Thereafter, each frozen tube containing the cryogel sample was chipped and immersed for 30 s in the solution of neodymium-containing contrasting agent (Section 4.1) preliminary cooled to −9 °C. Then, excess liquid from the chip’s surface was blown away with an air brush. The aluminum microscope stage of an EVO 10 scanning electron microscope (Carl Zeiss Microscopy GmbH, Jena, Germany) was cooled in liquid nitrogen as well, after which the tube with the sample was placed on the sample stage with the chip’s surface facing up. The morphology of the surface and the nearest subsurface layers of the samples was studied with a C2D detector in a low-vacuum mode (EP, 70 Pa) at an accelerating voltage of 20 kV. The average pore size of the PVA cryogels was determined using Image J software (version 1.54h) (National Institutes of Health, Bethesda, MD, USA) by measuring 50 randomly selected pores on SEM images.

#### 4.2.7. Release of Phenolic Additives from the Additive-Containing PVA Cryogels

PVA cryogels samples loaded with phenolic additives were formed in cylindrical molds; the volume of each gel sample was 1.7 mL. The cryogel was placed in a glass beaker filled with 10 mL of distilled water. The 100 μL portions of the liquid phase were further taken after definite time intervals, and the same volumes of pure water were returned to the system to compensate for the water phase. The aliquots were diluted with 2.9 mL of pure water, and optical absorbance was recorded using the VIS spectrum (T70 UV-VIS spectrophotometer, PG Instruments, Lutterworth, UK) on the suitable wavelength for every phenolic compound (pyrocatechol—277 nm; resorcinol—274 nm; hydroquinone—289 nm; phenol—270 nm). Then, the respective values of the concentration of phenolic additives were found from the preliminary obtained calibration graphs.

#### 4.2.8. In Vitro Evaluation of Antibacterial Activity of PVA Cryogels Carrying Additives of Phenol and Bis-Phenols

The evaluation of the antibacterial activity of the PVA cryogel samples loaded with phenolic additives was performed with the standard agar diffusion procedure [119]. The experiments were carried out using two test bacterial strains: *Staphylococcus aureus* (Gram-positive) and *Escherichia coli* (Gram-negative)—both from the collection of the Microbiology Department, Biology Faculty of M. V. Lomonosov, Moscow State University. Standard Petri dishes that contained agar nutrient medium (thickness of the medium layer was 4–5 mm) were dried at 37 °C for 40 min. The measurements of the growth inhibition zones (GIZs) for these bacterial strains were conducted after 24 h and 48 h of incubation at 30 °C. Statistically reliable results were obtained with a 5-fold repetition of the measurements for each series of samples.

## Figures and Tables

**Table 1:** Average pore size in the “secondary” cryogels derived from the additive-free and phenolic-additive-containing PVACGs.

SEM Image in Figure 6	Phenolic Additive in the “Primary” Cryogel	Average Pore Size in the “Secondary” Cryogel (μm)
**(a)**	Pyrocatechol	1.70 ± 0.24
**(b)**	Resorcinol	2.18 ± 0.30
**(c)**	Hydroquinone	2.77 ± 0.35
**(d)**	Phenol	4.02 ± 0.52
**(e)**	None	2.09 ± 0.27

**Table 2:** The values of GIZs formed upon 24 and 48 h incubation of the phenol-containing PVACGs onto the bacterial lawns of *S. aureus* and *E. coli.*

Phenolic Additive in the PVA Cryogel	Microorganism	GIZ (mm) after Incubation for	
		24 h	48 h
Pyrocatechol	*Staphylococcus* *aureus*	30 ± 1	32 ± 2
Resorcinol		64 ± 3	65 ± 3
Hydroquinone		26 ± 1	28 ± 2
Phenol		45 ± 2	48 ± 3
Pyrocatechol	*Escherichia* *coli*	-	-
Resorcinol		42 ± 3	54 ± 3
Hydroquinone		-	-
Phenol		39 ± 2	42 ± 2

## Data Availability

Data are contained within the article.
